# Discovery of the PARP1 Inhibitors from Natural Compounds Using Structure-Based Virtual Screening and Bioactivity Evaluation

**DOI:** 10.2174/0115734064350048241121110017

**Published:** 2025-02-07

**Authors:** Dabo Pan, Yaxuan Huang, Dewen Jiang, Xiaojie Jin, Mingkai Wu, Jianjun Luo, Yonghao Zhang

**Affiliations:** 1 Department of Medical Technology, Qiandongnan Vocational and Technical College for Nationalities, Kaili 556000, China;; 2 College of Pharmacy, Gansu University of Chinese Medicine, Lanzhou 730000, China;; 3 The Affiliated Dazu's Hospital of Chongqing Medical University, Dazu 402360, China

**Keywords:** PARP1 inhibitor, natural compound, demethyleneberberine, structure-based virtual screening, binding free energy calculation, molecular dynamics simulation

## Abstract

**Background:**

PARP1 (poly ADP-ribose polymerase 1, also known as ADPRT1) plays a significant role in DNA repair and has become an attractive target for treating PARP1-related diseases, such as cancer.

**Objective:**

This study aimed to discover inhibitors targeting PARP1 from the phytochemicals of Huangbai (*Phellodendron chinense* Schneid.), Baixianpi (*Dictamnus dasycarpus* Turcz.), and Shechuangzi (*Cnidium monnieri* (L.) Spreng.).

**Methods:**

The chemical compositions of Huangbai, Baixianpi, and Shechuangzi were extracted from the HERB database. Next, a combination of molecular docking and PARP1 enzyme assay was used to identify PARP1 inhibitors from these chemical components. Finally, molecular dynamics simulation and binding free energy calculation were used to explore the detailed interaction mode of these inhibitors with PARP1.

**Results:**

A total of 507 chemical constituents of Huangbai, Baixianpi, and Shechuangzi were collected from the HERB database. Four potential PARP1 inhibitors were screened based on molecular docking and PARP1 enzyme assay. Demethyleneberberine exhibited strong PARP1 inhibitory activity with an IC_50_ value of 2.0 ± 0.8 µM. The IC_50_ values of the inhibitory activities of 8-hydroxy dictanmnine, meranzin hydrate, and osthol on PARP1 ranged from 44 µM to 76 µM. Molecular dynamics simulation and binding free energy calculation suggested that the nonpolar interaction energies of HIS862, GLY863, TYR889, TYR896, PHE897, and TYR907 played a primary role in the binding of inhibitors to PARP1.

**Conclusion:**

Integrating molecular simulation and bioactivity testing was found to be an effective approach for the rapid discovery of targeted PARP1 inhibitors. Demethyleneberberine demonstrated strong PRAP1 inhibitory activity and has a good prospect for development.

## 1 INTRODUCTION

PARP1 is a multifunctional enzyme that plays a role in a number of cellular processes. One of its primary functions is to serve as a catalyst for the polymerisation of ADP-ribose units derived from NAD^+^, which results in the attachment of ADP-ribose to proteins [1]. PARP1 recruits ADP-ribose moieties to several amino acids, including arginine, The Affiliated Dazu's Hospital of Chongqing Medical University, Dazu 402360, China; E-mail: luojianjun@hospital.cqmu.edu.cn (Jianjun Luo) glutamate, aspartate, cysteine, lysine, and serine [2]. This process is crucial for the repair of damaged DNA and the remodeling of chromatin. PARP1 plays a pivotal role in this process, binding to chromatin or circRNA biogenesis and influencing its architectural structure, particularly in response to DNA damage [3, 4]. The PARP1-associated DNA repair pathways are classified into four types: base excision repair, non-homologous end joining, homologous recombination, and Okazaki fragment processing pathways [3, 5]. Furthermore, PARP1 has been implicated in various cellular pathways, such as regulating gene expression, autophagy, and apoptosis [6]. In conclusion, PARP1 is a versatile protein with diverse functions ranging from DNA repair and chromatin remodeling to gene regulation and cellular signaling. A comprehensive understanding of the multifaceted roles of PARP1 in diverse biological pathways, including anti-inflammatory [7, 8], immunomodulation [9], and metabolism [10], is essential for the development of targeted therapies and treatments for diseases, such as cancer.

PARP1 is composed of an N-terminal DNA binding domain, an auto modification domain, the tryptophan-glycine-arginine-rich domain, and a CAT domain [11]. The CAT domain comprises both HD and ART subdomains. The discovery of a small molecule capable of targeting the catalytic site on the interaction surface of these two subdomains has the potential to inhibit PARP1 activity. Currently, there are four FDA-approved PARP1 inhibitors, including Olaparib [12], Rucaparib [13], Niraparib [14], and Talazoparib [15, 16], and several inhibitors in preclinical and clinical studies [17-30]. Most PARP1 inhibitors contain benzamide structures, and they mimic the competitive binding of PARP1 by nicotinamide to NAD^+^ at the nicotinamide-ribose binding domain (NI site) of the PARP1 catalytic domain [31, 32]. Natural compounds are rich in structural diversity and a significant source of drug discovery. More than 32% of small molecule drugs marketed from 1981 to 2019 are derived from natural compounds [33]. Traditional Chinese medicines are rich in natural compounds with a wide range of pharmacological effects. Among them, Huangbai, Baixianpi, and Shechuangzi have been used for more than one thousand years, and they are known for their anti-tumor, anti-inflammatory, and antibacterial effects [34-36]. Integrating molecular modelling and bioactivity testing techniques to find targeted inhibitors from natural compounds is one of the rapid approaches [37-39].

In this study, we collected the chemical constituents of Huangbai, Baixianpi, and Shechuangzi and then obtained the potential PARP1 inhibitors using molecular docking. Four PARP1 candidate inhibitors were identified by PARP1 enzyme testing. Finally, molecular dynamics simulation, in conjunction with free energy calculation, was employed to elucidate the interactions between PARP1 and the candidate compound inhibitors from both structural and energetic perspectives. These candidate compounds are promising for the development of therapeutic targeting of PARP1-associated diseases.

## 2 METHODS AND MATERIALS

### 2.1 Collection of the Chemical Composition of Huangbai, Baixianpi, and Shechuangzi

A high-throughput experiment- and reference-guided database of traditional Chinese medicine (HERB) [40] was a relatively comprehensive database of ingredients of traditional Chinese medicines (TCM). The herb-related chemical compositions were collected from multiple TCM databases, including SymMap [41], TCMID [42], TCMSP [43], and TCM-ID [44]. The chemical compositions of Huangbai, Baixianpi, and Shechuangzi were screened using the herb names “Huangbai”, “Baixianpi,” and “Shechuangzi” in HERB, respectively. The 2D structures of chemical compositions were drawn based on their SMILE, ACS ID, or PubChem ID and then transferred to 3D structures using Chem3D software. These structures were prepared using the LigPrep module [45] in Schrödinger 2017 [46] using the OPLS-2005 force field [47]. The possible states were predicted at pH 7 ± 2 [48].

### 2.2 Structure-based Virtual Screening

The 3D structure of the PARP1-inhibitor complex was extracted from the Protein Data Bank (PDB ID: 6NRH [49]). The structures of PARP1 and inhibitor remained, and then all waters, SO_4_^2-^, and dimethyl sulfoxide were deleted. The PARP1-inhibitor complex was prepared, including adding hydrogen atoms, filling in missing loops, and minimizing structure using Protein Preparation Wizard. The binding site grid was 20*20*20 and identified in accordance with the ligand position by the Receptor Grid Generation module. The standard precision (SP) Glide module [50] was employed, docking the chemical compositions to the PARP1. These natural compounds with the top 50 molecular docking scores were selected, and the binary fingerprints were calculated using the canvas module [51]. These 50 compounds were classified into 4 categories according to their binary fingerprint properties. Compounds in each category with high docking scores and easily available for purchase were screened for PARP1 enzymatic assay.

### 2.3 *In vitro* PARP1 Enzymatic Assay

All chemical components were purchased from Chengdu Puruifa Co. The inhibitory activity of all tested chemical components against the PARP1 enzyme [52] was determined by ELISA in 96-well plates. A PBS buffer containing 10 mM NaH_2_PO_4_, 10 mM Na_2_HPO_4_, and 150 mM NaCl was prepared at pH 7.4. The wells were pre-coated with histones (20 μg/m L) diluted in 100 μL PBS buffer and incubated overnight at 4°C. Afterward, 100 μM NAD^+^ diluted in 30 μL reaction buffer (50 mM Tris, 2 mM MgCl_2_, pH 8.0), 25 μM biotinylated NAD^+^, and 200 nM sDNA were added to each well, and then 5 μL of compound or solvent control was added at varying concentrations. Then. 20 μL (5 ng) of PARP1 enzyme was added at 30°C, and 50 μL of HRP was added after 1 h. After 30 min of incubation, 100 μL of buffer (H_2_O_2_ and luminol in citrate buffer 0.1 M, pH 5.4) was added. The reaction was stopped, and the luminescent signal was measured using a multiwell spectrophotometer (Molecular Devices SpectraMax M5 microplate reader). The inhibition rate of PARP1 enzymatic activity was calculated as (Lu control − Lu treated/Lu control) × 100%. The concentration required for 50% inhibition of PARP1 enzymatic activity (IC_50_) was calculated using nonlinear regression with normalized dose-response fit using Prism GraphPad software. All activity tests were performed three times in parallel.

### 2.4 Molecular Dynamics (MD) Simulation and MM/GSA Free Energy Calculation

In order to obtain reasonable force field parameters of a small molecule, the small molecule was optimized at the HF/6-31G* level, and then its atomic charge was calculated using Gaussian09 software. The parameters of the force field and atomic charges of small molecules were generated by GAFF [53] and RESP fitting [54] in AmberTools22 [55], respectively. The AMBER14 force field [56] and TIP3P water [57] were used for proteins and water molecules, respectively. The total charge of the PRAP1-ligand complex system was 0 due to 5 or 6 Cl^-^ ions being added to the corresponding system. A 10 Å rectangular water box was added to each system. Detailed molecular dynamics simulations were performed as described previously [58, 59]. The system simulated 5000 steps of restraint energy minimization (0.1 kcal/mol•Å^2^) and unrestraint energy minimization, respectively. Next, the system was heated from 0 K to 310 K with a force constant of 2 kcal/mol•Å^2^ on the complex. Finally, 500 ns MD simulation was performed under the NPT ensemble (temperature 310 K, pressure 1 atm) without any restraint. The Molecular Mechanics Generalized Born Surface Area (MM/GBSA) method [60] was used to calculate the binding free energy between the ligand and PARP1. One thousand snapshots (one snapshot taken every 500 ps) and 100 snapshots (one snapshot taken every 5 ns) structures were used for enthalpy and entropy calculations, respectively. To elucidate the interaction mode between the ligand and PARP1, we decomposed the binding free energy (only the enthalpic term) to each amino acid residue.

## 3 RESULTS AND DISCUSSION

### 3.1 The Chemical Composition of Huangbai, Baixianpi, and Shechuangzi

**Fig. (1):**
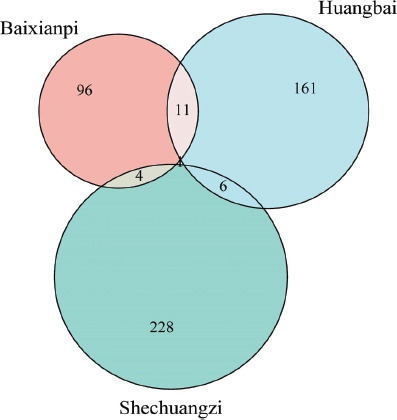
The Venn diagram illustrates the common chemical compositions of Huangbai, Baixianpi, and Shechuangzi.

In the HERB database, 179, 112, and 239 chemical components were obtained by using the herb keywords “Huangbai, Baixianpi, and Shechuangzi”, respectively (Fig. **1**). As shown in Fig. (**1**), compound beta-sitosterol (HBIN018278) was present in all three plants. Furthermore, two plants, Baixianpi and Shechuangzi, contained four common chemical constituents: beta-pinene (HBIN018242), copaene (HBIN021422), Uvadex (HBIN047654), and (Z)-caryophyllene (HBIN048842). Six common components in Huangbai and Shechuangzi were beta-elemene (HBIN018094), myrcene (HBIN036067), oleic acid (HBIN038026), palmitic acid (HBIN038680), poriferast-5-en-3-beta-ol (HBIN040583), and stigmasterol (HBIN044918). There were 11 common components in Huangbai and Baixianpi, including acetic acid (HBIN014399), campesteryl ferulate (HBIN019484), dictamine (HBIN023700), fagarine (HBIN026335), limonin (HBIN033253), obacunoic acid (HBIN037640), obacunone (HBIN037642), ptelein (HBIN041152), quercetin (HBIN041495), sitogluside (HBIN044152), and skimmianin (HBIN044179). These common components were likely to be the material basis for three herbs to exert the same kind of medicinal effects. Beta-sitosterol was a common component in three herbs and possessed various biological actions, such as antioxidant, anticancer, anti-diabetic, antimicrobial, and immunomodulatory activities [61]. Beta-pinene, a common component in Baixianpi and Shechuangzi, has a wide range of pharmacological activities, including antimicrobial, antimalarial, antioxidant, and anti-inflammatory [62]. Removal of the chemical components common in the three plants yielded 507 chemical components.

### 3.2 The Candidate Compounds by Virtual Screening

For the rapid discovery of potential PARP1 inhibitors from these chemical components, molecular docking is one of the better approaches. Molecular docking enables rapid determination of small molecule-protein or protein-protein matches in terms of geometry and charge to obtain potential small molecules or proteins (peptides) with high affinity for the target [38, 39, 63-68]. Here, the SP Glide module was used for the docking of these chemical components with PARP1. The top 50 molecules in molecular docking scoring were extracted and are shown in Table **S1**. Based on the fingerprint information of the chemical components, 50 chemical components were clustered into 4 categories. Taking into account the structural diversity and accessibility of the chemical components, five chemical components were selected for subsequent activity testing (Table **1**).

### 3.3 The Candidate Compounds PARP1 Enzymatic Activity

**Fig. (2):**
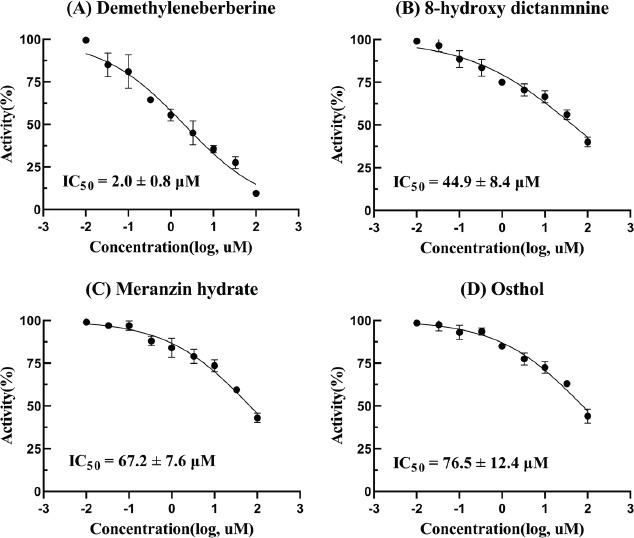
The IC_50_ values of candidate compounds for PARP1 inhibition, (**A**) Demethyleneberberine, (**B**) 8-hydroxy dictanmnine, (**C**) Meranzin hydrate, (**D**) Osthol. (All data were obtained by triple testing, ± standard deviation).

The inhibition of PARP1 by the five candidate compounds at a concentration of 30 µM was first tested (Table **1**). As mentioned in Table **1**, Phellavin_qt (HBIN039429) inhibited PARP1 activity only 3.0 ± 1.2% at a concentration of 30 µM. However, the other four candidate compounds, demethyleneberberine (HBIN023247), 8-hydroxy dictanmnine (HBIN010138), meranzin hydrate (HBIN034757), and osthol (HBIN038387), showed 75.2 ± 5.7%, 28.4 ± 3.4%, 22.8 ± 4.9%, and 20.1 ± 7.1% inhibition of PARP1 at 30 µM concentration, respectively. Next, we further evaluated the IC_50_ values of these four candidate compounds for PARP1 inhibition (Fig. **2**).

Inhibition of PARP1 by four candidate compounds indicated concentration dependence. Among four candidate compounds, demethyleneberberine had the strongest ability to inhibit PARP1 with an IC_50_ value of 2.0 ± 0.8 µM (Fig. **2A**). Compared to demethyleneberberine, 8-hydroxy dictanmnine demonstrated weaker inhibition of PARP1 with an IC_50_ value of 44.9 ± 8.4 µM (Fig. **2B**). Meranzin hydrate and osthol showed similar ability to inhibit PARP1 with IC_50_ values of 67.2 ± 7.6 µM and 76.5 ± 12.4 µM, respectively (Figs. **2C** and **2D**). In order to further explore the action mechanism of these candidate compounds with PARP1, we combined techniques, such as molecular dynamics simulation and binding free energy, to elucidate them from both energetic and structural perspectives.

### 3.4 Interaction Mechanisms Between Candidate Compounds and PARP1

**Fig. (3):**
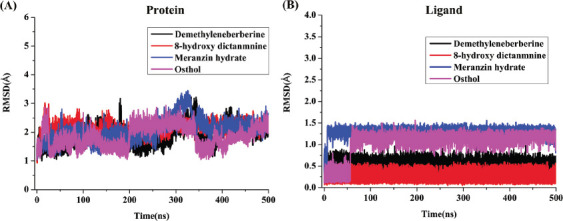
Time evolution of RMSD of Cα atoms for the residues of protein (**A**) and the heavy atoms of candidate compounds (**B**).

In order to assess whether the simulated system reached equilibrium, we examined the changes in the simulated system protein and small molecule RMSDs over time (Fig. **3**). In Fig. **3A**, it could be seen that the RMSD of proteins fluctuates between 1 and 3 during the 500 ns simulation for the four simulated systems. Interestingly, the RMSDs of the heavy atoms of 4 ligands fluctuated even less, fluctuating between 0 and 1.5 with respect to each other (Fig. **3B**). This suggested that all simulated systems were relatively stable and tended to reach equilibrium throughout the simulation.

Next, the binding free energy between the candidate compounds and PARP1 was calculated using the MM-GBSA method. As shown in Table **2**, the binding free energies of demethyleneberberine, 8-hydroxy dictanmnine, meranzin hydrate, and osthol to PARP1 were -12.0 kcal/mol, -9.1 kcal/mol, -6.2 kcal/mol, and -5.6 kcal/mol, respectively, which was consistent with the experimental results that these four candidate compounds inhibited the enzymatic activity of PARP1. Among all energy terms, in PARP1-Demethyleneberberine complex, the non-polar interaction term (ΔGnonpolar), polar interaction term (ΔGpolar), and entropy term (T∆S) were -31.3 kcal/mol, 2.9 kcal/mol, and -16.4 kcal/mol, respectively. This suggested that the polar interaction term and entropy were unfavorable for demethyleneberberine binding to PARP1, while the non-polar interaction term favored demethyleneberberine binding to PARP1. Interestingly, the binding of the other three candidate compounds to PARP1 showed similar results compared to demethyleneberberine. The non-polar interaction term plays a significant role in the binding between the candidate compound and PARP1.

**Fig. (4):**
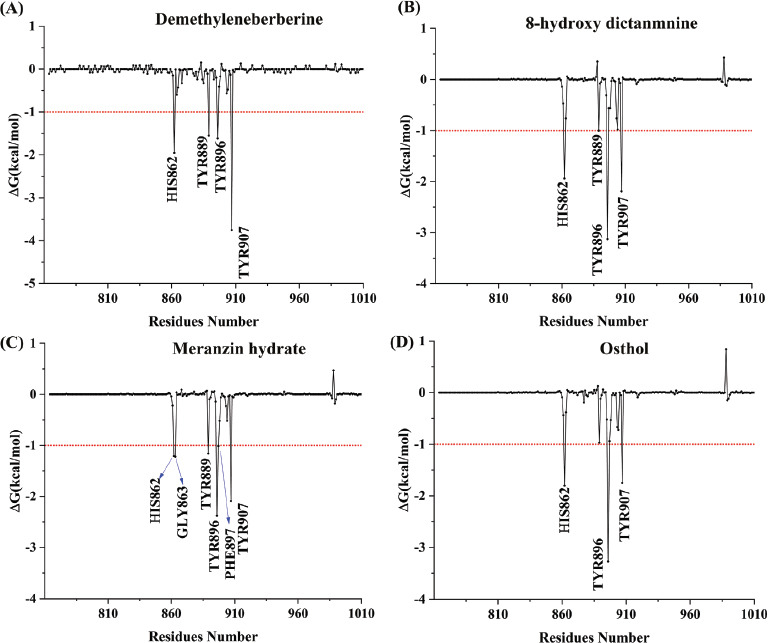
The decomposition energy of amino acids, (**A**) PARP1-Demethyleneberberine complex, (**B**) PARP1-8-hydroxy dictanmnine complex, (**C**) PARP1-Meranzin hydrate complex, (**D**) PARP1-Osthol complex.

Furthermore, in order to find the amino acids that play a primary role in the binding between the candidate compound and PARP1, we decomposed the binding free energy to each amino acid residue (Fig. **4**). Amino acid with binding free energy contribution values better than -1 kcal/mol was defined as the critical amino acid. There were four critical amino acids, including HIS862, TYR889, TYR896, and TYR907, in the PARP1-Demethyleneberberine complex (Fig. **4A**). The binding free energy contribution of TYR907 was the highest value (-3.8 kcal/mol). In comparison, the binding free energy contribution of TYR889 was relatively weak (-1.6 kcal/mol). There were four, six, and three critical amino acids in the PARP1-8-hydroxy dictanmnine complex, PARP1-Meranzin hydrate complex, and PARP1-Osthol complex, respectively (Figs. **4A**-**D**). Surprisingly, in the four candidate compounds-PARP1complexes, these key amino acids were mainly concentrated in 6 amino acid residues, including HIS862, GLY863, TYR889, TYR896, PHE897, and TYR907. These six amino acids played a major role in binding to PARP1 and were also detected in other PARP1 inhibitors-PARP1 complex [69-73].

The decomposition energy terms of six key amino acids were extracted and are shown in Fig. (**5**). The energetic contributions of six key amino acids differed in different systems, while the trend that the nonpolar interaction energy contributes more than the polar interaction energy in the binding of candidate compounds to PARP1 was consistent. In PARP1-Demethyleneberberine complex, the nonpolar energy terms of HIS862, TYR889, TYR896, and TYR907 were -1.9 kcal/mol, -1.5 kcal/mol, -1.8 kcal/mol, and-4.3 kcal/mol, respectively. However, the polar energy terms of HIS862, TYR889, TYR896, and TYR907 were -0.1 kcal/mol, -0.1 kcal/mol, 0.2 kcal/mol, and 0.6 kcal/mol, respectively. Similarly, the nonpolar interaction energies of TYR896 in PARP1-8-hydroxy dictanmnine complex, PARP1-Meranzin hydrate complex, and PARP1-Osthol complex reached -3.3 kcal/mol, -3.3 kcal/mol, and -4.2 kcal/mol, respectively. In contrast, their polar interaction energies in the corresponding systems were only 0.2 kcal/mol, 1.0 kcal/mol, and 0.9 kcal/mol. To visualize the interaction mode between the candidate compounds and PARP1, the average structures of the 500 ns trajectory were extracted and are shown in Fig. (**6**).

**Fig. (6):**
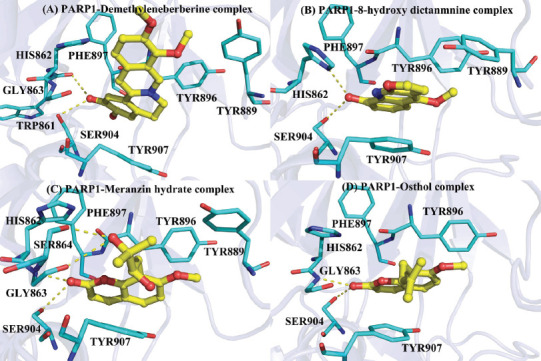
The interactions between PARP1 and candidate compounds, (**A**) PARP1-Demethyleneberberine complex, (**B**) PARP1-8-hydroxy dictanmnine complex, (**C**) PARP1-Meranzin hydrate complex, (**D**) PARP1-Osthol complex.

As shown in . (**6A**), the hydroxyl group of demethyleneberberine was inserted into the bottom of the active pocket of PARP1 and formed two hydrogen bonds with SER904 and GLY863. In addition, demethyleneberberine formed a strong π-π stacking interaction with TYR907, PHE897, and TYR896. There were two hydrogen bonds between the hydroxyl group of 8-hydroxy dictanmnine and PARP1. The strong π-π stacking interaction between 8-hydroxy dictanmnine and several aromatic amino acids, including TYR889, TYR896, and TYR907, was determined (Fig. **6B**). Meranzin hydrate was structurally similar to Osthol, and their binding models to PARP1 were also similar. They formed a π-π stacking interaction with TYR907, PHE897, and TYR896 of PARP1. Meranzin hydrate formed four hydrogen bonds with PARP1 due to the presence of the hydroxyl groups in the side chain. On the other hand, Osthol only formed two hydrogen bonds with PARP1 (Figs. **6C** and **6D**).

## CONCLUSION

We employed molecular docking, bioactivity testing, molecular dynamics simulation, and binding free energy calculation to identify four candidate compounds, including demethyleneberberine, 8-hydroxy dictanmnine, meranzin hydrate, and osthol targeting PARP1 from the chemical compositions of Huangbai, Baixianpi, and Shechuangzi. Furthermore, the detailed interaction mode between these compounds and PARP1 was elucidated, providing lead compounds and some references for further design of PARP1 inhibitors.

## AUTHORS’ CONTRIBUTIONS

Their authors confirm their contribution to the paper as follows: JD, HY, JX, and WM were responsible for taking charge of the computer-based drug screening and original draft. Corresponding authors PD, LJ, and ZY contributed to the conception and design of this study and were also responsible for revising the manuscript. Others were responsible for revising the manuscript. All authors reviewed the results and approved the final version of the manuscript.

## Figures and Tables

**Fig. (5):**
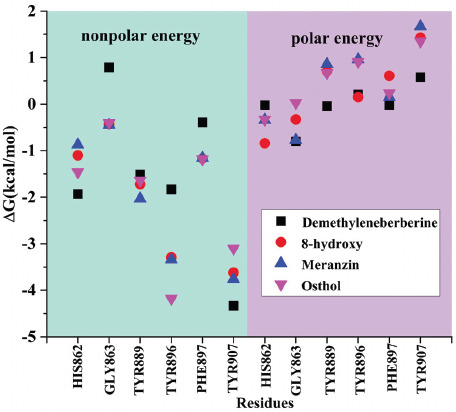
The decomposition binding free energy terms of the key amino acids.

**Table 1:** Chemical characteristics and *in vitro* activities of virtual screening hits against PARP1.

**No.**	**HERB ID**	**Ingredient Name**	**Herb**	**Docking Score (kcal/mol)**	**Structure Cluster**	**Inhibition % (30 µM)a**	**Structure**
1	HBIN039429	Phellavin_qt	Huangbai	-7.9	1	3.0 ± 1.2	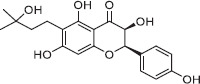
2	HBIN023247	demethyleneberberine	Huangbai	-7.8	2	75.2 ± 5.7	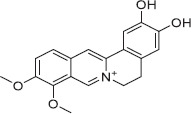
3	HBIN010138	8-hydroxy dictanmnine	Baixianpi	-7.6	3	28.4 ± 3.4	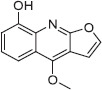
4	HBIN034757	meranzin hydrate	Shechuangzi	-7.9	4	22.8 ± 4.9	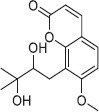
5	HBIN038387	osthol	Shechuangzi	-7.8	4	20.1 ± 7.1	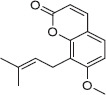

**Note:** All data were obtained by triple testing (± standard deviation).

**Table 2:** The calculated binding free energy between PARP1 and candidate compounds (kcal/mol).

	**ΔE_ele_**	**ΔE_vdw_**	**ΔG_sol-np_**	**ΔG_sol-ele_**	**ΔG_nonpolar_^b^**	**ΔG_polar_^a^**	**ΔH_bind_**	**T∆S**	**ΔG_bind_**	**IC_50_ (μM)**
Demethyleneberberine	-29.2	-26.5	-4.8	32.1	-31.3	2.9	-28.4	-16.4	-12.0	2.0 ± 0.8
8-hydroxy dictanmnine	-9.5	-30.2	-3.9	20.4	-34.1	10.9	-23.3	-14.1	-9.1	44.9 ± 8.4
Meranzin hydrate	-18.1	-34.5	-4.9	36.2	-39.4	18.1	-21.3	-15.1	-6.2	67.2 ± 7.6
Osthol	-15.1	-28.9	-4.4	27.2	-33.3	12.1	-21.2	-15.7	-5.6	76.5 ± 12.4

**Note: **^a^ ΔG_polar_ a =ΔE_ele_ +ΔG_sol-ele_; ^b^ ΔGnonpolar =ΔE_vdw_ +ΔG_sol-np_.

## Data Availability

All data generated or analysed during this study are included in this published article.

## LIST OF ABBREVIATIONS

PARP1: Poly ADP-ribose Polymerase 1
TCM: Traditional Chinese Medicines
HERB: High-throughput Experiment- and Reference-guided Database
SP: Standard Precision

## References

[1] Ray C.A., Nussenzweig A. (2017). The multifaceted roles of PARP1 in DNA repair and chromatin remodelling.. Nat. Rev. Mol. Cell Biol..

[2] Alemasova E.E., Lavrik O.I. (2019). Poly(ADP-ribosyl)ation by PARP1: Reaction mechanism and regulatory proteins.. Nucleic Acids Res..

[3] Spiegel J.O., Van Houten B., Durrant J.D. (2021). PARP1: Structural insights and pharmacological targets for inhibition.. DNA Repair (Amst.).

[4] Dhahri H., Fondufe-Mittendorf Y.N. (2024). Exploring the interplay between PARP1 and circRNA biogenesis and function.. Wiley Interdiscip. Rev. RNA.

[5] Deeksha W., Rajakumara E. (2024). Regulatory apoptotic fragment of PARP1 complements catalytic fragment for PAR and DNA-dependent activity but inhibits DNA-induced catalytic stimulation of PARP2.. DNA Repair (Amst.).

[6] Xiao G., Lundine D., Annor G.K., Canar J., Ellison V., Polotskaia A., Donabedian P.L., Reiner T., Khramtsova G.F., Olopade O.I., Mazo A., Bargonetti J. (2020). Gain-of-function mutant p53 R273H interacts with replicating DNA and PARP1 in breast cancer.. Cancer Res..

[7] Krug S., Gupta M., Kumar P., Feller L., Ihms E.A., Kang B.G., Srikrishna G., Dawson T.M., Dawson V.L., Bishai W.R. (2023). Inhibition of host PARP1 contributes to the anti-inflammatory and antitubercular activity of pyrazinamide.. Nat. Commun..

[8] Wen J.J., Dejesus J.E., Radhakrishnan G.L., Radhakrishnan R.S. (2023). PARP1 inhibition and effect on burn injury-induced inflammatory response and cardiac function.. J. Am. Coll. Surg..

[9] Böhi F., Hottiger M.O. (2024). Expanding the perspective on PARP1 and its inhibitors in cancer therapy: From DNA damage repair to immunomodulation.. Biomedicines.

[10] Ke Y., Wang C., Zhang J., Zhong X., Wang R., Zeng X., Ba X. (2019). The role of PARPs in inflammation-and metabolic-related diseases: Molecular mechanisms and beyond.. Cells.

[11] Langelier M.F., Planck J.L., Roy S., Pascal J.M. (2012). Structural basis for DNA damage-dependent poly(ADP-ribosyl)ation by human PARP-1.. Science.

[12] Menear K.A., Adcock C., Boulter R., Cockcroft X., Copsey L., Cranston A., Dillon K.J., Drzewiecki J., Garman S., Gomez S., Javaid H., Kerrigan F., Knights C., Lau A., Loh V.M., Matthews I.T.W., Moore S., O’Connor M.J., Smith G.C.M., Martin N.M.B. (2008). 4-[3-(4-cyclopropanecarbonylpiperazine-1-carbonyl)-4-fluorobenzyl]-2H-phthalazin-1-one: A novel bioavailable inhibitor of poly(ADP-ribose) polymerase-1.. J. Med. Chem..

[13] Thomas H.D., Calabrese C.R., Batey M.A., Canan S., Hostomsky Z., Kyle S., Maegley K.A., Newell D.R., Skalitzky D., Wang L.Z., Webber S.E., Curtin N.J. (2007). Preclinical selection of a novel poly(ADP-ribose) polymerase inhibitor for clinical trial.. Mol. Cancer Ther..

[14] Jones P., Altamura S., Boueres J., Ferrigno F., Fonsi M., Giomini C., Lamartina S., Monteagudo E., Ontoria J.M., Orsale M.V., Palumbi M.C., Pesci S., Roscilli G., Scarpelli R., Schultz-Fademrecht C., Toniatti C., Rowley M. (2009). Discovery of 2-{4-[(3S)-piperidin-3-yl]phenyl}-2H-indazole-7-carboxamide (MK-4827): A novel oral poly(ADP-ribose)polymerase (PARP) inhibitor efficacious in BRCA-1 and -2 mutant tumors.. J. Med. Chem..

[15] Litton J.K., Rugo H.S., Ettl J., Hurvitz S.A., Gonçalves A., Lee K.H., Fehrenbacher L., Yerushalmi R., Mina L.A., Martin M., Roché H., Im Y.H., Quek R.G.W., Markova D., Tudor I.C., Hannah A.L., Eiermann W., Blum J.L. (2018). Talazoparib in patients with advanced breast cancer and a germline *BRCA* mutation.. N. Engl. J. Med..

[16] Agarwal N., Azad A.A., Carles J., Fay A.P., Matsubara N., Heinrich D., Szczylik C., De Giorgi U., Young Joung J., Fong P.C.C., Voog E., Jones R.J., Shore N.D., Dunshee C., Zschäbitz S., Oldenburg J., Lin X., Healy C.G., Di Santo N., Zohren F., Fizazi K. (2023). Talazoparib plus enzalutamide in men with first-line metastatic castration-resistant prostate cancer (TALAPRO-2): A randomised, placebo-controlled, phase 3 trial.. Lancet.

[17] Yuan B., Ye N., Song S.S., Wang Y.T., Song Z., Chen H.D., Chen C.H., Huan X.J., Wang Y.Q., Su Y., Shen Y.Y., Sun Y.M., Yang X.Y., Chen Y., Guo S.Y., Gan Y., Gao Z.W., Chen X.Y., Ding J., He J.X., Zhang A., Miao Z.H. (2017). Poly(ADP-ribose)polymerase (PARP) inhibition and anticancer activity of simmiparib, a new inhibitor undergoing clinical trials.. Cancer Lett..

[18] Donawho C.K., Luo Y., Luo Y., Penning T.D., Bauch J.L., Bouska J.J., Bontcheva-Diaz V.D., Cox B.F., DeWeese T.L., Dillehay L.E., Ferguson D.C., Ghoreishi-Haack N.S., Grimm D.R., Guan R., Han E.K., Holley-Shanks R.R., Hristov B., Idler K.B., Jarvis K., Johnson E.F., Kleinberg L.R., Klinghofer V., Lasko L.M., Liu X., Marsh K.C., McGonigal T.P., Meulbroek J.A., Olson A.M., Palma J.P., Rodriguez L.E., Shi Y., Stavropoulos J.A., Tsurutani A.C., Zhu G.D., Rosenberg S.H., Giranda V.L., Frost D.J. (2007). ABT-888, an orally active poly(ADP-ribose) polymerase inhibitor that potentiates DNA-damaging agents in preclinical tumor models.. Clin. Cancer Res..

[19] Li X., Wang C., Li S., Yin F., Luo H., Zhang Y., Luo Z., Chen Y., Wan S., Kong L., Wang X. (2024). Dual target PARP1/EZH2 inhibitors inducing excessive autophagy and producing synthetic lethality for triple-negative breast cancer therapy.. Eur. J. Med. Chem..

[20] Zhang L., Zhen Y., Feng L., Li Z., Lu Y., Wang G., Ouyang L. (2023). Discovery of a novel dual-target inhibitor of CDK12 and PARP1 that induces synthetic lethality for treatment of triple-negative breast cancer.. Eur. J. Med. Chem..

[21] Zhang J., Zhang J., Li H., Chen L., Yao D. (2023). Dual-target inhibitors of PARP1 in cancer therapy: A drug discovery perspective.. Drug Discov. Today.

[22] Wang P., Zhu W.T., Wang Y., Song S.S., Xi Y., Yang X.Y., Shen Y.Y., Su Y., Sun Y.M., Gao Y.L., Chen Y., Ding J., Miao Z.H., Zhang A., He J.X. (2023). Identification of [1,2,4]Triazolo[4,3-a]pyrazine PARP1 inhibitors with overcome acquired resistance activities.. Eur. J. Med. Chem..

[23] Kim C., Wang X.D., Jang S., Yu Y. (2023). PARP1 inhibitors induce pyroptosis *via* caspase 3-mediated gasdermin E cleavage.. Biochem. Biophys. Res. Commun..

[24] Kellett T., Noor R., Zhou Q., Esquer H., Sala R., Stojanovic P., Rudolph J., Luger K., LaBarbera D.V. (2023). HTS discovery of PARP1-HPF1 complex inhibitors in cancer.. SLAS Discov..

[25] Peng X., Pan W., Jiang F., Chen W., Qi Z., Peng W., Chen J. (2022). Selective PARP1 inhibitors, PARP1-based dual-target inhibitors, PROTAC PARP1 degraders, and prodrugs of PARP1 inhibitors for cancer therapy.. Pharmacol. Res..

[26] Liu X., Wei X., Li X., Yu R., Jiang T., Zhao C. (2022). Design, synthesis, and bioactivity study on Lissodendrins B derivatives as PARP1 inhibitor.. Bioorg. Med. Chem..

[27] Li G., Lin S., Yu Z., Wu X., Liu J., Tu G., Liu Q., Tang Y., Jiang Q., Xu J., Huang Q., Wu L.A. (2022). PARP1 PROTAC as a novel strategy against PARP inhibitor resistance *via* promotion of ferroptosis in p53-positive breast cancer.. Biochem. Pharmacol..

[28] Kayumov M., Jia L., Pardaev A., Song S.S., Mirzaakhmedov S., Ding C., Cheng Y.J., Zhang R.I., Bao X., Miao Z.H., He J.X., Zhang A. (2022). Design, synthesis and pharmacological evaluation of new PARP1 inhibitors by merging pharmacophores of olaparib and the natural product alantolactone.. Eur. J. Med. Chem..

[29] Eldin A., Osman E., Hanafy N.S., George R.F., El-Moghazy S.M., El-Moghazy S.M. (2020). Design and synthesis of some barbituric and 1,3-dimethylbarbituric acid derivatives: A non-classical scaffold for potential PARP1 inhibitors.. Bioorg. Chem..

[30] Jain P.G., Patel B.D. (2019). Medicinal chemistry approaches of poly ADP-Ribose polymerase 1 (PARP1) inhibitors as anticancer agents - A recent update.. Eur. J. Med. Chem..

[31] Langelier M.F., Zandarashvili L., Aguiar P.M., Black B.E., Pascal J.M. (2018). NAD+ analog reveals PARP-1 substrate-blocking mechanism and allosteric communication from catalytic center to DNA-binding domains.. Nat. Commun..

[32] Thorsell A.G., Ekblad T., Karlberg T., Löw M., Pinto A.F., Trésaugues L., Moche M., Cohen M.S., Schüler H. (2017). Structural basis for potency and promiscuity in poly(ADP-ribose) polymerase (PARP) and tankyrase inhibitors.. J. Med. Chem..

[33] Newman D.J., Cragg G.M. (2020). Natural products as sources of new drugs over the nearly four decades from 01/1981 to 09/2019.. J. Nat. Prod..

[34] Sun Y., Lenon G.B., Yang A.W.H. (2019). Phellodendri cortex: A phytochemical, pharmacological, and pharmacokinetic review.. Evid. Based Complement. Alternat. Med..

[35] Qin Y., Quan H.F., Zhou X.R., Chen S.J., Xia W.X., Li H., Huang H.L., Fu X.Y., Dong L. (2021). The traditional uses, phytochemistry, pharmacology and toxicology of *Dictamnus dasycarpus*: A review.. J. Pharm. Pharmacol..

[36] Li Y.M., Jia M., Li H.Q., Zhang N.D., Wen X., Rahman K., Zhang Q.Y., Qin L.P. (2015). *Cnidium monnieri*: A review of traditional uses, phytochemical and ethnopharmacological properties.. Am. J. Chin. Med..

[37] Asiamah I., Obiri S.A., Tamekloe W., Armah F.A., Borquaye L.S. (2023). Applications of molecular docking in natural products-based drug discovery.. Sci. Am..

[38] Kaur T., Madgulkar A., Bhalekar M., Asgaonkar K. (2019). Molecular docking in formulation and development.. Curr. Drug Discov. Technol..

[39] Saikia S., Bordoloi M. (2019). Molecular docking: Challenges, advances and its use in drug discovery perspective.. Curr. Drug Targets.

[40] Fang S., Dong L., Liu L., Guo J., Zhao L., Zhang J., Bu D., Liu X., Huo P., Cao W., Dong Q., Wu J., Zeng X., Wu Y., Zhao Y. (2021). HERB: a high-throughput experiment- and reference-guided database of traditional Chinese medicine.. Nucleic Acids Res..

[41] Wu Y., Zhang F., Yang K., Fang S., Bu D., Li H., Sun L., Hu H., Gao K., Wang W., Zhou X., Zhao Y., Chen J. (2019). SymMap: An integrative database of traditional Chinese medicine enhanced by symptom mapping.. Nucleic Acids Res..

[42] Huang L., Xie D., Yu Y., Liu H., Shi Y., Shi T., Wen C. (2018). TCMID 2.0: A comprehensive resource for TCM.. Nucleic Acids Res..

[43] Ru J., Li P., Wang J., Zhou W., Li B., Huang C., Li P., Guo Z., Tao W., Yang Y., Xu X., Li Y., Wang Y., Yang L. (2014). TCMSP: A database of systems pharmacology for drug discovery from herbal medicines.. J. Cheminform..

[44] Wang J., Zhou H., Han L., Chen X., Chen Y., Cao Z. (2005). Traditional Chinese medicine information database.. Clin. Pharmacol. Ther..

[45] (2017). Schrödinger Release 2017-4: LigPrep. https://www.sciepub.com/reference/350859.

[46] Schrodinger Release 2017-3: LigPrep.. https://scirp.org/reference/referencespapers?referenceid=3115697.

[47] Kaminski G.A., Friesner R.A., Tirado-Rives J., Jorgensen W.L. (2001). Evaluation and reparametrization of the OPLS-AA force field for proteins *via* comparison with accurate quantum chemical calculations on peptides.. J. Phys. Chem. B.

[48] (2017). Epik, version 2.0. https://www.schrodinger.com/platform/products/epik/.

[49] Velagapudi U.K., Langelier M.F., Delgado-Martin C., Diolaiti M.E., Bakker S., Ashworth A., Patel B.A., Shao X., Pascal J.M., Talele T.T. (2019). Design and synthesis of poly(ADP-ribose) polymerase inhibitors: Impact of adenosine pocket-binding motif appendage to the 3-oxo-2,3-dihydrobenzofuran-7-carboxamide on potency and selectivity.. J. Med. Chem..

[50] Friesner R.A., Banks J.L., Murphy R.B., Halgren T.A., Klicic J.J., Mainz D.T., Repasky M.P., Knoll E.H., Shelley M., Perry J.K., Shaw D.E., Francis P., Shenkin P.S. (2004). Glide: A new approach for rapid, accurate docking and scoring. 1. Method and assessment of docking accuracy.. J. Med. Chem..

[51] (2020). Schrödinger release 2017-3: Canvas.. https://www.schrodinger.com/canvas.

[52] Ye N., Chen C.H., Chen T., Song Z., He J.X., Huan X.J., Song S.S., Liu Q., Chen Y., Ding J., Xu Y., Miao Z.H., Zhang A. (2013). Design, synthesis, and biological evaluation of a series of benzo[de][1,7]naphthyridin-7(8H)-ones bearing a functionalized longer chain appendage as novel PARP1 inhibitors.. J. Med. Chem..

[53] Wang J., Wolf R.M., Caldwell J.W., Kollman P.A., Case D.A. (2004). Development and testing of a general amber force field.. J. Comput. Chem..

[54] Bayly C.I., Cieplak P., Cornell W., Kollman P.A. (1993). A well-behaved electrostatic potential based method using charge restraints for deriving atomic charges: The RESP model.. J. Phys. Chem..

[55] Salomon-Ferrer R., Case D.A., Walker R.C. (2013). An overview of the Amber biomolecular simulation package.. Wiley Interdiscip. Rev. Comput. Mol. Sci..

[56] Maier J.A., Martinez C., Kasavajhala K., Wickstrom L., Hauser K.E., Simmerling C. (2015). ff14SB: Improving the accuracy of protein side chain and backbone parameters from ff99SB.. J. Chem. Theory Comput..

[57] Onufriev A.V., Izadi S. (2018). Water models for biomolecular simulations.. Wiley Interdiscip. Rev. Comput. Mol. Sci..

[58] Pan D., Huang Y., Jiang D., Zhang Y., Wu M., Han M., Jin X. (2024). Discovery of an EP300 inhibitor using structure-based virtual screening and bioactivity evaluation.. Curr. Pharm. Des..

[59] Pan D., Zeng C., Zhang W., Li T., Qin Z., Yao X., Dai Y., Yao Z., Yu Y., Yao X. (2019). Non-volatile pungent compounds isolated from *Zingiber officinale* and their mechanisms of action.. Food Funct..

[60] Miller B.R., McGee T.D., Swails J.M., Homeyer N., Gohlke H., Roitberg A.E. (2012). *MMPBSA.py*: An efficient program for end-state free energy calculations.. J. Chem. Theory Comput..

[61] Babu S., Jayaraman S. (2020). An update on β-sitosterol: A potential herbal nutraceutical for diabetic management.. Biomed. Pharmacother..

[62] Salehi B., Upadhyay S., Erdogan Orhan I., Kumar Jugran A. (2019). Therapeutic potential of α- and β-pinene: A miracle gift of nature.. Biomolecules.

[63] Vidal-Limon A., Aguilar-Toalá J.E., Liceaga A.M. (2022). Integration of molecular docking analysis and molecular dynamics simulations for studying food proteins and bioactive peptides.. J. Agric. Food Chem..

[64] Li H., Sun X., Cui W., Xu M., Dong J., Ekundayo B.E., Ni D., Rao Z., Guo L., Stahlberg H., Yuan S., Vogel H. (2024). Computational drug development for membrane protein targets.. Nat. Biotechnol..

[65] Santos L.H.S., Ferreira R.S., Caffarena E.R. (2019). Integrating molecular docking and molecular dynamics simulations.. Methods Mol. Biol..

[66] Ferreira L., Dos Santos R., Oliva G., Andricopulo A. (2015). Molecular docking and structure-based drug design strategies.. Molecules.

[67] Stanzione F., Giangreco I., Cole J.C. (2021). Use of molecular docking computational tools in drug discovery.. Prog. Med. Chem..

[68] Agu P.C., Afiukwa C.A., Orji O.U., Ezeh E.M., Ofoke I.H., Ogbu C.O., Ugwuja E.I., Aja P.M. (2023). Molecular docking as a tool for the discovery of molecular targets of nutraceuticals in diseases management.. Sci. Rep..

[69] Khizer H., Maryam A., Ansari A., Ahmad M.S., Khalid R.R. (2024). Leveraging shape screening and molecular dynamics simulations to optimize PARP1-Specific chemo/radio-potentiators for antitumor drug design.. Arch. Biochem. Biophys..

[70] Zhao J., Yu N., Zhao X., Quan W., Shu M. (2023). 3D-QSAR, molecular docking, and molecular dynamics analysis of dihydrodiazaindolone derivatives as PARP-1 inhibitors.. J. Mol. Model..

[71] Nilov D., Maluchenko N., Kurgina T., Pushkarev S., Lys A., Kutuzov M., Gerasimova N., Feofanov A., Švedas V., Lavrik O., Studitsky V.M. (2020). Molecular mechanisms of PARP-1 inhibitor 7-methylguanine.. Int. J. Mol. Sci..

[72] Kinoshita T., Nakanishi I., Warizaya M., Iwashita A., Kido Y., Hattori K., Fujii T. (2004). Inhibitor‐induced structural change of the active site of human poly(ADP‐ribose) polymerase.. FEBS Lett..

[73] McCarthy K.A., Marcotte D.J., Parelkar S., McKinnon C.L., Trammell L.E., Stangeland E.L., Jetson R.R. (2024). Discovery of potent isoindolinone inhibitors that target an active conformation of PARP1 using DNA‐encoded libraries.. ChemMedChem.

