# Supplementary material for: Discovery of the PARP1 Inhibitors from Natural Compounds Using Structure-Based Virtual Screening and Bioactivity Evaluation
Source: Med Chem. 2025 Feb 7;22(1):57–66. doi: 10.2174/0115734064350048241121110017 (PMC13284649; doi:10.2174/0115734064350048241121110017)
Supplement: Supplementary file 1 [file MC-22-1-57_SD1.pdf]

Supplementary Materials

Discovery of the PARP1 inhibitors from natural compounds using structure-based virtual screening and bioactivity evaluation

Dabo Pan[1,*] Yaxuan Huang[1] Dewen Jiang[1] Xiaojie Jin[2] Mingkai Wu[1] Jianjun Luo[3,*] Yonghao Zhang[1,*]

[1]Department of Medical Technology, Qiandongnan Vocational and Technical College for Nationalities, Kaili 556000, China; [2]College of Pharmacy, Gansu University of Chinese Medicine, Lanzhou 730000, China; [3]The Affiliated Dazu's Hospital of Chongqing Medical University, Dazu 402360, China

Table S1. Chemical composition information with the top 50 molecular docking scoring.

NO.	Ingredient ID	Ingredient Name	Herb	CAS ID	Docking score (kcal/mol)	Cluster
1	HBIN039424	phellamurin_qt	Huangbai	52589-11-4	-8.71	1
2	HBIN006701	(2S,3S)-3,5,7-trihydroxy-2-(4-hydroxyphenyl)chroman-4-one	Huangbai	480-20-6	-8.19	1
3	HBIN009367	3'-O-methyl taxifolin	Baixianpi	-	-8.19	1
4	HBIN026335	Fagarine	Huangbai, Baixianpi	524-15-2	-8.11	1
5	HBIN039429	Phellavin_qt	Huangbai	-	-7.86	1
6	HBIN039428	phellavin	Huangbai	32507-67-8	-7.76	1
7	HBIN023247	demethyleneberberine	Huangbai	25459-91-0	-7.82	2
8	HBIN046198	thalifendine	Huangbai	18207-71-1	-7.68	2
9	HBIN017897	berberrubine	Huangbai	15401-69-1	-7.67	2
10	HBIN017893	berberine	Huangbai	2086-83-1	-7.60	2
11	HBIN031501	jatrorrizine	Huangbai	3621-38-3	-7.58	2
12	HBIN030613	Isocorypalmine	Huangbai	483-34-1	-8.33	3
13	HBIN044961	STOCK1N-14407	Huangbai	3520-14-7	-7.63	3
14	HBIN010138	8-hydroxy dictanmnine	Baixianpi	2255-50-7	-7.57	3
15	HBIN022782	Dauricine (8CI)	Huangbai	474-58-8	-7.57	3
16	HBIN046315	THM	Baixianpi	35902-13-7	-9.99	4
17	HBIN029831	hyperin	Huangbai	-	-9.10	4
18	HBIN019614	Canthin-6-one	Huangbai	479-43-6	-8.67	4
19	HBIN023182	delta7-Dehydrosophoramine	Huangbai	-	-8.65	4
20	HBIN030780	isogosferol	Shechuangzi	53319-52-1	-8.56	4
21	HBIN037372	Noroxyhydrastinine	Huangbai	21796-14-5	-8.48	4
22	HBIN015924	Amurensin_qt	Huangbai	641-94-1	-8.44	4
23	HBIN029419	Hirsutrin	Baixianpi	21637-25-2	-8.35	4
24	HBIN048464	xanthotoxol	Shechuangzi	2009-24-7	-8.35	4
25	HBIN033803	luteolin	Baixianpi	491-70-3	-8.32	4
26	HBIN041121	psoralen	Baixianpi	66-97-7	-8.25	4

1573-4064/25 © 2025 Bentham Science Publishers

27	HBIN015916	Amurensin	Huangbai	641-94-1	-8.23	4
28	HBIN037087	N-Methylflindersine	Huangbai	50333-13-6	-8.15	4
29	HBIN021172	cnidimol B	Shechuangzi	103629-81-8	-8.08	4
30	HBIN013897	(8S)-8-(2-hydroxypropan-2-yl)-8,9-dihydrofuro[2,3-h]chromen-2-one	Shechuangzi	3804-70-4	-8.04	4
31	HBIN041495	quercetin	Huangbai, Baixianpi	117-39-5	-8.03	4
32	HBIN040654	Prangenidin	Shechuangzi	642-05-7	-8.02	4
33	HBIN030539	Isobutyryl shikonin	Shechuangzi	-	-8.01	4
34	HBIN005130	2'-Acetylangelicin	Shechuangzi	25152-84-5	-8.00	4
35	HBIN038459	Oxophorone	Huangbai	1125-21-9	-7.99	4
36	HBIN048372	wogonin	Baixianpi	632-85-9	-7.96	4
37	HBIN034757	meranzin hydrate	Shechuangzi	5875-49-0	-7.93	4
38	HBIN024184	Diosmetin	Shechuangzi	520-34-3	-7.92	4
39	HBIN034297	Majudin	Shechuangzi	484-20-8	-7.89	4
40	HBIN030913	Isomaculosidine	Baixianpi	518-96-7	-7.88	4
41	HBIN032446	Kvannin	Shechuangzi	1760-27-6	-7.84	4
42	HBIN038387	osthol	Shechuangzi	484-12-8	-7.82	4
43	HBIN003013	[(1R)-1-(5,8-dihydroxy-1,4-dioxo-2-naphthyl)-4-methyl-pent-3-enyl] 3-methylbutanoate	Shechuangzi	52387-14-1	-7.81	4
44	HBIN021176	cnidimol F	Shechuangzi	144050-04-4	-7.80	4
45	HBIN013317	7-Methoxy-4-methylcoumarin	Shechuangzi	2555-28-4	-7.79	4
46	HBIN013311	7-methoxy-2-oxochromene-8-carbaldehyde	Shechuangzi	6724-42-1	-7.74	4
47	HBIN016087	angelicin	Shechuangzi	523-50-2	-7.72	4
48	HBIN047654	Uvadex	Shechuangzi, Baixianpi	12692-94-3	-7.72	4
49	HBIN017367	auraptenol	Shechuangzi	1221-43-8	-7.67	4
50	HBIN042657	rutaecarpine	Huangbai	84-26-4	-7.65	4
